# Haplotype specific-sequencing reveals *MBL2 *association with asymptomatic *Plasmodium falciparum *infection

**DOI:** 10.1186/1475-2875-8-97

**Published:** 2009-05-11

**Authors:** Angelica BW Boldt, Iara J Messias-Reason, Bertrand Lell, Saadou Issifou, Maria Lucia Alves Pedroso, Peter G Kremsner, Jürgen FJ Kun

**Affiliations:** 1 Department of Parasitology, Institute of Tropical Medicine, University of Tübingen, Wilhelmstrasse 27, 72074 Tübingen, Germany; 2 Laboratory of Molecular Immunopathology, Hospital de Clínicas, Federal University of Paraná, Curitiba, Brazil; 3 Medical Research Unit, Albert Schweitzer Hospital, Lambaréné, Gabon

## Abstract

**Background:**

Mannose binding lectin (MBL) has an important role in the activation of the complement system and opsonization of pathogenic microorganisms. Frequent polymorphisms found in the *MBL2 *gene affect the concentration and functionality of the protein and are associated with enhanced susceptibility to severe malaria in African children. Most *MBL2 *typing strategies were designed to the analysis of selected variants, the significance of whole haplotypes is poorly known. In this work, a new typing strategy was developed and validated in an association analysis of *MBL2 *with adult asymptomatic infection.

**Methods:**

*MBL2 *allele-specific fragments of 144 healthy Gabonese adults were amplified by using haplotype-specific sequencing (HSS), a new strategy that combines sequence-specific PCR and sequence-based typing. The Gabonese were investigated for the presence of *Plasmodium falciparum *parasitaemia by the amplification of parasite genes, immunochromatographic antigen detection and microscopic analysis. HSS results were also compared with a previously used real-time PCR (RT-PCR) method in 72 Euro-Brazilians.

**Results:**

Fourteen polymorphisms were identified beside the commonly investigated promoter (*H, L*; *X, Y*; *P, Q*) and exon 1 (*A, O*; *O *= *B*, *C *or *D*) variants. The MBL2*LYPA/LYPA genotype was associated with the absence of asymptomatic infection (P = 0.017), whereas the MBL2*LYQC haplotype and *YA/YO *+ *YO/YO *genotypes were associated with positive parasite counts in asymptomatic adults (P = 0.033 and 0.018, respectively). The associations were specific to *LYPA *(identical to the reference sequence Y16577) and *LYQC *(Y16578) and would not have been revealed by standard genotyping, as there was no association with *LYPA *and *LYQC *haplotypes carrying new polymorphisms defined by sequence-based typing. In contrast, HSS and RT-PCR produced very similar results in the less diverse European-derived population.

**Conclusion:**

In this work, a new typing strategy for a highly polymorphic gene was developed and validated focusing on the asymptomatic status of *P. falciparum-*infected adults. In populations with high nucleotide diversity, it allowed for the identification of associations with fine-scaled haplotypes that would not have been found using common typing techniques. In this preliminary study, *MBL2 *haplotypes or SNPs linked to them were found associated with susceptibility to infection and parasitaemia control of asymptomatic adults.

## Background

*Plasmodium falciparum *infection causes between one and two million deaths annually, mostly in African children less than 5 years of age. This means that every 30 seconds a child dies of malaria. Those who survive became increasingly immune to severe malaria with each disease episode [1]. Although the immune mechanisms in older individuals clear a large proportion of infected erythrocytes, part of the parasites can persist in the circulation without causing symptoms. Semi-immunity seems to be dependent on frequent *P. falciparum *reinfections and can be lost within a few weeks if the individual leaves the endemic area [2]. Frequent reinfections nevertheless are associated with anaemia, secondary bacterial infections and hyper-reactive malarial splenomegaly. Individuals with asymptomatic parasitaemia may also serve as a reservoir for transmissible parasites [3].

Several genetic polymorphisms that modulate immune response were found to be associated with protection against malaria. They include alleles of the HLA system [4], of tumour necrosis factor (*TNF*) (for a review, see [5]), nitric oxide synthase 2 (*NOS2A*) [6] and mannose-binding lectin (*MBL2*) [7,8]. Mannose-binding lectin recognizes sugar moieties such as mannose and N-acetyl-D-glucosamine on a wide range of different microorganisms [9], and on parasites such as *Schistosoma mansoni *and *P. falciparum *[10,11]. Upon binding, it activates the complement system via interaction with MBL-associated serine proteases (MASP-1, -2, -3 and Map19) and kills the potential pathogen by the membrane-attack complex or by complement-mediated phagocytosis through increased deposition of opsonic C3 fragments. MBL is also able to directly opsonize the microorganism for phagocytosis and to modulate the release of pro-inflammatory cytokines.

*MBL2 *deficiency is associated with the susceptibility and severity of many diseases, as well as with protection against intracellular infections as tuberculosis, leprosy and leishmaniasis [12-14]. Three single nucleotide polymorphisms (SNPs) in the first exon of the gene: MBL2*D (*Arg52Cys*), *B *(*Gly54Asp*) and *C *(*Gly57Glu*) [15,16] are mainly responsible for the reduction of circulating levels of MBL oligomers and of functional activity of the protein, which is very common and widespread in the human species. They have been collectively labeled *O*, whereas the major alleles at these loci have been called *A*. The concentration of the protein in serum is further modulated by at least three SNPs in the promoter region: MBL2*H, L (located 550 bp before the transcription start site), *X, Y *(located 221 bp before the transcription start site) and *P, Q *(not coding SNP located 4 bp after the transcription start site) [17,18]. Linkage disequilibrium between the SNPs is responsible for eight haplotypes associated with increasingly lower MBL serum concentration: MBL2*HYPA = *LYQA *= *LYPA *> *LXPA *>> *HYPD *= *LYPB *= *LYQC *= *LYPD *[7]. Due to the strong concentration-lowering effect of the *X/Y *and *A/O *exon 1 variants, *HYPA*, *LYQA *and *LYPA *are commonly evaluated as a joined diplotype group "*YA*". Accordingly, *LXPA *is analyzed as "*XA*" and *HYPD*, *LYPB*, *LYQC *and *LYPD *as "*YO*". This approach nevertheless neglects important hitch-hiking effects that become evident with the analysis of complete haplotypes, as well as the functional effects of new SNPs. E.g. 14 additional allelic haplotypes were recently defined by this group, most of them similar to *LYQA *and *LYPA*, and one of them was found associated with severe malaria [7].

This new *MBL2 *typing strategy for physical separation and sequencing analysis of promoter-exon 1 haplotypes, was validated by comparing it with real-time PCR (RT-PCR) in 72 Brazilian subjects of another study [19] and focusing for the first time on adult asymptomatic *P. falciparum *infection among 144 Gabonese individuals. The typing strategy was called haplotype-specific sequencing (HSS) and showed higher cost-benefit for physical separation of small-sized haplotypes if compared with cloning [20] and haplotype-specific extraction (HSE) methods [21]. It also allowed for the identification of associations with fine-scaled haplotypes that would not have been found using other typing techniques in an Afro-derived population.

## Methods

### Subjects and samples

A total of 144 Gabonese adults that took part of a large epidemiologic survey to detect the prevalence of asymptomatic *Plasmodium spp*. infection in the villages around Lambaréné, Gabon, were investigated. There were three individuals with *P. malariae*, negative for *P. falciparum*, in the original study [22]. They were not included in this study. Seventy-two Euro-Brazilians (Brazilians with major European ancestry), previously genotyped using RT-PCR with fluorescent hybridisation probes [19], were also genotyped with HSS to compare typing strategies. They were healthy blood donors resident in Paraná state, South Brazil, sampled for different association studies. Ethical clearance was obtained from the ethics committee of the International Foundation Albert Schweitzer Hospital and from the local medical ethics committee in Brazil.

### Parasitaemia detection

At least two experienced technicians independently counted the parasites in thick blood smears (TBS). They also performed a rapid diagnostic test (RDT) to detect markers for all four human pathogenic *Plasmodium *species (NOW^® ^ICT Malaria Test; Binax, Inc., Portland, ME) and amplified subtelomeric variable open reading frame (*stevor*)genes to find submicroscopic parasitaemia by PCR. The amplification of *stevor *genes is highly sensitive, allowing the detection of as less as 10 parasites in 1 ml of blood [23]. Detailed description of the procedures is described elsewhere [22]. Although PCR is taken as the gold standard for detecting asymptomatic parasitaemia, sequestered parasites cannot be detected. Yet the RDT test relies on the detection of histidine-rich protein 2 (HRP-2), a protein secreted by the parasite. Due to the long half-life of HRP-2, a positive RDT result can also be interpreted as evidence for an infection cleared weeks before sampling. A positive RDT result was found for six of the PCR negative adults. Since there could have been a symptomatic parasitaemia in the recent past of these individuals, they were excluded from the non-parasitized group and not included in the asymptomatic group.

Thus the PCR negative group consisted of 62 individuals (mean age, 30 ± 8 years [range, 18–49 years]) and the PCR positive asymptomatic group, of 76 individuals (mean age, 28 ± 7 years [range, 18–47 years]). All individuals with a positive TBS result were PCR positive as well. Nevertheless 78% of asymptomatic parasitized individuals would not have been found using only the TBS test. The same could be stated for 45% of them using only the RDT test. These figures are in agreement with a larger study (78% and 48%, respectively) [22].

### *MBL2 *typing

Blood was collected with the anticoagulant ethylenediaminetetraacetic acid and DNA was extracted from peripheral blood mononuclear cells through standard salting-out and phenol/chloroform/isoamyl alcohol methods. A fragment of 1059 nucleotides was amplified using the forward primers MBLfor (5'-ATGGGGCTAGGCTGCTGAG-3') and the reverse primer MBLrev (5'-CCAACACGTACCTGGTTCCC-3'). Sequence specific (SSP) PCR products were generated using the same reverse primer, combined to forward primers specific for variant *H *(Hf: 5'-GCTTACCCAGGCAAGCCTGTG-3') or for the variant *L *(Lf: 5'-GCTTACCCAGGCAAGCCTGTC-3'); for the variant *X *(Xf: 5'-CCATTTGTTCTCACTGCCACC-3') or for the variant *Y *(Yf: 5'-CCATTTGTTCTCACTGCCACG-3'). The PCR product achieved with the primers Hf or Lf and MBLrev and Xf or Yf and MBLrev were 837 and 508 nucleotides in length, respectively. Hf and Lf were also combined to specific reverse primers for the variant *P *(Pr: 5'-CTCAGTTAATGAACACATATTTACCG-3') or for the variant *Q *(Qr: 5'-CTCAGTTAATGAACACATATTTACCA-3'), generating a product of 599 nucleotides. All fragments were sequenced with the amplification primers or with an internal exon 1 sequencing primer, MBLint (5'-GAGGCCAGGGATGGGTCATC-3'), using Big dye terminator version 1.1 chemistry (Applied Biosystems, Foster City, CA). Amplification conditions are described in detail elsewhere [24]. The reactions were purified with the Performa DTR V3 system (Edge BioSystems, Gaithersburg, MD) and analysed on an automated sequencer (ABI Prism 3100 Genetic Analyzer, Applied Biosystems, Foster City, CA). New variants (singletons) were verified by reamplification and resequencing.

### Statistical analyses

Genotype and haplotype frequencies were obtained by direct counting. The haplotype frequency distributions found in this and in other studies [7,25] were compared by applying the exact test of population differentiation of Raymond and Rousset [26]. Deviations from Hardy-Weinberg equilibrium were tested using the approach described by Guo and Thompson [27]. These tests were performed using the software package ARLEQUIN version 3.1 [28]. Possible associations between *MBL2 *genotypes/alleles and susceptibility to asymptomatic infection were analysed with Fisher's exact tests.

## Results

**Figure 1:**
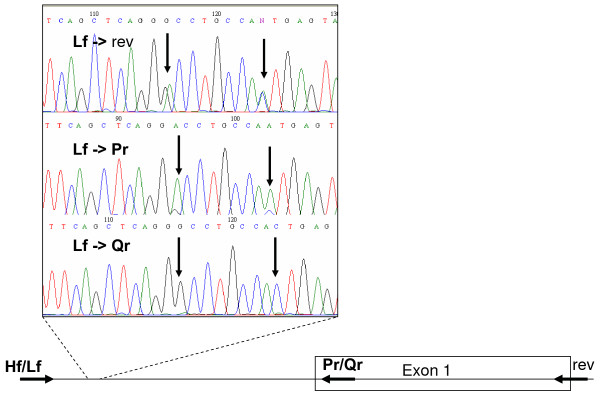
**Haplotyping by PCR-SSP fragments sequencing**. Results are from a *LYPA/LYQA *sample. Both chromosomes were coamplified with the Lf and the generic rev primer (rev270 from [7]), and separately amplified using Pr or Qr as reverse SSP primers. Vertical arrows in the electropherograms show the *g.388G>A *(rs7100749) and *g.396A>C *(rs11003124) SNPs. The approximate sites of the primers and of the sequences are represented by horizontal arrows and traced lines, respectively. In bold: SSP primers.

The same results were achieved using RT-PCR [19] and HSS in the 72 Euro-Brazilian samples, except for one individual, haplotyped as *LYQA *using real-time and as *LYPA *using this approach. Two heterozygote individuals also presented unexpected SNPs, which were only revealed by sequencing: *g.388G>A *(in the *LYPAs1 *haplotype) and *g.797C>A *(in the *LYQCs1 *haplotype). In the Gabonese, 14 polymorphisms were identified beside the commonly investigated promoter (*H, L*; *X, Y*; *P, Q*) and exon 1 (*A, O*; *O *= *B*, *C *or *D*) variants. In contrast to RT-PCR, HSS did not rely on maximum likelihood algorithms for haplotyping, but amplified and sequenced allelic fragments of heterozygote individuals in different tubes to determine the phase of all SNPs (Figure 1).

*MBL2 *haplotypes identified in this study are listed [see Additional file 1]. The haplotype distribution of the Gabonese adults was homogeneous with those of the previously investigated Gabonese schoolchildren, children with uncomplicated malaria and with severe malaria [7] and of Ghanaian children [25]. On average, 38% of the Gabonese genotypes carried a *LYPA-, LYQA- *or *LYQC *haplotype presenting other SNPs than the commonly investigated *H, L; X, Y; P, Q *and *A, O *variants. They represent 20% of all haplotypes, which conventionally were called *LYPAs1, LYPAs2, LYPAs3, LYQAs1, LYQAs2, LYQCs1, LYQCs2, LYQCs3 *(s for "similar" to the mentioned haplotype). The haplotype with the most frequent *O *variant in Africa – MBL2*LYQC – was associated with a positive parasite count [14% or 17/124 in non-parasitized vs. 29% or 10/34 in TBS positive individuals, P = 0.033, O.R. = 2.62 (1.1–6.4)]. This association was specific to the *LYQC *haplotype (identical to the reference sequence Y16578).

Genotype frequencies from both Gabonese groups were at Hardy and Weinberg equilibrium (table 1). There were five *LYPA/LYPA *homozygotes in the adult non-parasitized group but none in the asymptomatic group (8% or 5/62 vs. 0% or 0/76, P = 0.017). This association was specific to *LYPA *(identical to the reference sequence Y16577): frequencies of all other genotypes with *LYPA-*similar haplotypes presented no significant differences. To look for the effect of *X, Y *and *A, O *variants, known to modulate the concentration of high-order MBL oligomers, the genotypes were grouped as: *YA/YA, YA/XA, XA/XA, XA/YO, YA/YO *and *YO/YO *(table 1). In agreement with the *LYQC *association, there was a significant increase in the frequency of *YO/YO *and *YA/YO *genotypes between parasite-free and asymptomatic parasitized adults having a positive TBS result [(27% or 17/62 vs. 59% or 10/17, P = 0.018, O.R. = 3.8 (1.2–11.5)].

**Table 1:** *MBL2 *genotype association with parasitaemia status

	**PCR-**	**PCR+**	**PCR+TBS+**			**PCR-**	**PCR+**	**PCR+TBS+**	
**Genotype**	**n = 62**	**n = 76**	**n = 17**	**Parasite mia**	**Genotype**	**n = 62**	**n = 76**	**n = 17**	**Parasite mia**

*LXPA/LXPA*	2	1	0	0	*LXPA/LYQC*	5	7	0	0

*XA/XA*	2	1	0	0	*LXPA/LYQCs1*	0	2	0	0

*LXPA/LYPA*	1	1	0	0	*LXPA/LYQCs3*	1	0	0	-

*LXPA/LYPAs1*	0	3	0	0	*XA/YO*	7	9	0	0

*LXPA/LYPAs2*	0	1	0	0	*HYPA/LYPB*	1	0	0	-

*LXPA/LYQA*	6	3	1	0 (0–3800)	*HYPA/LYQC*	2	3	2	26 (26–165)

*LXPA/LYQAs2*	1	2	1	0–94	*LYPA/LYPB*	0	1	0	0

*XA/YA*	8	10	2	94 (0–3800)	*LYPA/LYQC*	1	2	0	0

*HYPA/LYPA*	1	2	1	0–45	*LYPA/LYQCs1*	1	0	0	-

*HYPA/LYQA*	2	1	0	0	*LYPAs1/LYQC*	2	1	1	380

***LYPA/LYPA***	**5**	**0**	0	**-**	*LYPAs1/LYQCs1*	0	2	1	0–2700

*LYPA/LYPAs1*	1	3	0	0	*LYPB/LYQA*	0	2	0	0

*LYPA/LYQA*	4	7	2	33 (0–38)	*LYPB/LYQAs2*	1	1	0	0

*LYPA/LYQAs2*	2	4	0	0	*LYQA/LYQC*	4	3	2	12 (0–47)

*LYPAs1/LYPAs1*	1	1	0	0	*LYQA/LYQCs1*	1	1	0	0

*LYPAs1/LYQA*	2	2	0	0	*LYQA/LYQCs2*	1	0	0	-

*LYPAs2/LYQA*	1	0	0	-	*LYQAs2/LYQC*	1	2	2	33–340

*LYPAs3/LYQA*	0	1	0	-	*YA/YO*	15	18	8	106 (0–2700)

*LYQA/LYQA*	5	7	1	0 (0–1200)	*LYPB/LYQC*	0	1	0	0

*LYQA/LYQAs1*	1	0	0	-	*LYQC/LYQC*	1	4	1	0 (0–514)

*LYQA/LYQAs2*	3	2	0	0	*LYQC/LYQCs3*	0	1	1	16650

*LYQAs2/LYQAs2*	0	1	0	0	*LYQCs1/LYQCs3*	1	0	0	-

*LYPF/LYQA*	0	1	1	183	*YO/YO*	2	6	2	514 (0–16650)

*YA/YA*	28	32	5	38 (0–1200)	***YA/YO + YO/YO***	**17**	24	**10**	174 (0–16650)

*LXPA/LYPB*	1	0	0	-					

The median values for parasite counts are given, followed by the range in parentheses. Parasitaemia was calculated as parasites per microliter (counted parasites/no. of microscopic fields) × 600 [41]. The final result was obtained by calculating the arithmetic mean from parasite count of the first and second readings [22]. In bold: significant difference.

## Discussion

There are numerous published *MBL2 *genotyping techniques, based on restriction fragment length polymorphisms (RFLP) of PCR products [29], sequence-specific PCR [30,31], denaturing gradient gel electrophoresis of PCR-amplified fragments [32], real-time PCR with the hybridization of sequence-specific probes [19] and sequence-based typing [33]. Most of the earliest techniques are restricted to the identification of the conventional *H/L, X/Y, P/Q *and *A/B/C/D *variants and thus of the *HYPA *(reference sequence: Y16581), *HYPD *(Y16582), *LXPA *(Y16580), *LYPA *(Y16577), *LYPB *(Y16579), *LYQA *(Y16576) and *LYQC *(Y16578) haplotypes. Newer methods that allow the identification of other SNPs rely on maximum likelihood methods for phasing. It was established in previous work [7] that around 35% of all *LYPA *haplotypes in the Gabonese population are not identical with the reference sequence Y16577. These haplotypes carry additional variants, the most frequent being *g.388G>A*. Around 17% of the *LYQA *haplotypes are not identical with Y16576 and around 13% of the *LYQC *haplotypes are not identical with Y16578 in the same population. In contrast, only 1.4% of the Euro-Brazilian group presented uncommon SNPs. This is mainly due to the higher heterozygosity and nucleotide diversity of the *MBL2 *promoter region in African, compared to non-African populations [34]. In order not to lose information, it is recommended to use of a sequence-based typing technique in *MBL2 *disease association studies with Afro-derived populations.

Phase knowledge also minimizes errors due to haplotyping algorithms. This could be achieved using cloning or haplotype-specific extraction (HSE). Both offer the possibility of isolating large fragments, even the whole homologue chromosomes of heterozygote individuals [20]. Cloning is nevertheless time-consuming and requires expertise with living cells and much more bench work than HSE, which relies on magnetic beads for haplotype separation [21]. After haplotype separation, HSE allows for amplification of genomic haplotype DNA, followed by further downstream applications. Small-sized haplotypes as those formed by the *MBL2 *promoter-exon1 SNPs and indels are more readily analyzed with HSS, which unifies physical separation, amplification and partial genotyping through SSP primers in the same reaction. HSS principles have also been applied to HLA genes before [35,36].

*MBL2 *physical haplotyping with SSP primers began in 2002 and led to the discovery of *LYPD*, a previously unrecognized *MBL2 *haplotype [30], and to the conclusion that the linkage disequilibrium of the *D *variant with the *HYP *promoter was not absolute, as formerly assumed. In contrast, other haplotypes whose existence was predicted by statistical algorithms have physically not been confirmed, including *HXPA *and *LYQB *[34].

This work is the first *MBL2 *association study including only truly non-parasitized and asymptomatic *P. falciparum *infected adults. In contrast, other authors evaluated the association of *O *alleles with asymptomatic infection in children. The long-lasting asymptomatic status of infected African children depends on the number of previous malaria attacks [37]. This could explain why there was no association of *O *alleles with asymptomatic infection in a follow-up study with 158 Gabonese schoolchildren [38], but in a recent study with 480 Ghanaians, which included PCR-detected asymptomatic individuals [25]. A reason for absence of association in the first study could also be the inclusion of submicroscopically parasitized individuals as healthy controls, as well as false heterozygosity results because of incomplete digestion of the PCR product by the enzymes used in the *MBL2 *genotyping method [32].

Asymptomatic *P. falciparum *infection is highly prevalent in the Gabonese adult population [22]. It seems to be a *sine qua non *condition for the maintenance of semi-immunity, which is lost within some weeks outside an endemic area [2]. It could nevertheless also lead to anaemia and other secondary complications. In this work, a significant association of the *LYPA/LYPA *genotype with protection against asymptomatic parasitaemia was found. None of the *LYPA-*similar haplotypes were associated with the infection, and so the association would not have been revealed by conventional *MBL2 *typing techniques. *LYPA *is probably an ancient haplotype, with a wide geographical distribution. It occurred in nine different African groups, being less common in East Asian and rare in Amerindian(-similar) populations [24,34]. In the Gabonese population, approximately half of the *LYPA *(Y16577) haplotypes carry a linked SNP (*-1165 G>T*), which could actually be responsible for the observed association. This SNP was only found in African populations [34]. It is less likely that the association is due to a 3' variant, since the 5' promoter-exon 1 region is separated from the 3' region by a recombination hot spot [39].

There was also an association of the MBL2*LYQC variant, *YO/YO *and *YA/YO *genotypes with microscopically detectable parasites. These preliminary findings in adults correlate well with some studies in children. In Ghanaian infected children, the *YO/YO *genotype was found to be associated with higher parasitaemia [40] and grouped *LYQC *haplotypesas well as *A/O *genotypes, with higher susceptibility to *P. falciparum *infection [25]. Despite some similarities between the associations found in adults and in children, the role of MBL in the immune response against *P. falciparum *may also change with the acquisition of semi immunity as individuals grow older, depending on the frequency of disease episodes. The replication of the results in a larger adult setting, as well as functional *in vitro *and *in vivo *studies, would help to clarify this suggestion.

## Conclusion

HSS was validated as a fast and highly informative method for analyzing small-sized haplotypes of polymorphic genes in populations with high nucleotide diversity, as are the vast majority of Afro-derived ethnic groups. It is more affordable than HSE, which relies on magnetic beads, and as cloning, which requires living cells. In this preliminary study, *MBL2 *haplotypes or SNPs linked to them were associated with the susceptibility to infection and with parasitaemia control of asymptomatic adults.

## Competing interests

The authors declare that they have no competing interests.

## Authors' contributions

ABWB developed the HSS method, performed the *MBL2 *genotyping and statistical analysis, and drafted the manuscript. IJM-R helped in interpretation of the data and to draft the manuscript. MLAP, SI and BL collected the Euro-Brazilian and Gabonese samples, respectively. PGK participated in the design of the study and revised the manuscript for important intellectual content. JFJK coordinated the study, participated in the statistical analysis and helped to draft the manuscript. All authors read and approved the final manuscript.

## Supplementary Material

Additional file 1 ***MBL2 *nucleotide changes and haplotype frequencies in the Gabonese samples according to parasitaemia status**. The data show the haplotype frequency in Gabonese individuals with and without detected parasites. The positions corresponding to the SNPs and to the deletion (position of the first deleted nucleotide) are shown in the first row (Reference sequence: Y16577). In bold: significant different frequencies. *LYPAs1, LYPAs2 *and *LYPAs3*:*LYPA*-similar haplotypes; *LYQAs1 *and *LYQAs2*: *LYQA-*similar haplotypes; *LYQCs1, LYQCs2 *and *LYQCs3*:*LYQC*-similar haplotypes. PCR polymerase chain reaction, TBS thick blood smear, – negative result, + positive result positive result, N number of chromosomes. All PCR-individuals were also negative in a Rapid Diagnosis Test. In SNP database: *g.273G>C *as rs11003125, *g.388G>A *as rs7100749, *g.396A>C *as rs11003124, *g.456G>T *as rs35615810, *g.474A>G *as rs7084554, *g.478G>A *as ss107796301, *g.482A>G *as ss107796302, *g.487A>G *as rs36014597, *g.495delAAAGAG *as rs10556764, *g.578G>A *as rs35236971, *g.602G>C *as rs7096206, *g.659C>T *as ss107796304, *g.712A>T *as ss107796305, *g.753C>T *as rs11003123, *g.797C>A *as rs45602536, *g.826C>T *as rs7095891, *g.925C>G *as ss107796306, *g.926T>G *as ss107796307, *g.1052G>A *as rs1800450, *g.1061G>A *as rs1800451. All ss numbers were submitted by the authors to the SNP database and will be changed to rs numbers in the near future. Click here for file
