# Supplementary material for: Haplotype specific-sequencing reveals MBL2 association with asymptomatic Plasmodium falciparum infection
Source: Malar J. 2009 May 11;8:97. doi: 10.1186/1475-2875-8-97 (PMC2689254; doi:10.1186/1475-2875-8-97)
Supplement: Additional file 1 — MBL2 nucleotide changes and haplotype frequencies in the Gabonese samples according to parasitaemia status. The data show the haplotype frequency in Gabonese individuals with and without detected parasites. The positions corresponding to the SNPs and to the deletion (position of the first deleted nucleotide) are shown in the first row (Reference sequence: Y16577). In bold: significant different frequencies. LYPAs1, LYPAs2 and LYPAs3:LYPA-similar haplotypes; LYQAs1 and LYQAs2: LYQA-similar haplotypes; LYQCs1, LYQCs2 and LYQCs3:LYQC-similar haplotypes. PCR polymerase chain reaction, TBS thick blood smear, – negative result, + positive result positive result, N number of chromosomes. All PCR-individuals were also negative in a Rapid Diagnosis Test. In SNP database: g.273G>C as rs11003125, g.388G>A as rs7100749, g.396A>C as rs11003124, g.456G>T as rs35615810, g.474A>G as rs7084554, g.478G>A as ss107796301, g.482A>G as ss107796302, g.487A>G as rs36014597, g.495delAAAGAG as rs10556764, g.578G>A as rs35236971, g.602G>C as rs7096206, g.659C>T as ss107796304, g.712A>T as ss107796305, g.753C>T as rs11003123, g.797C>A as rs45602536, g.826C>T as rs7095891, g.925C>G as ss107796306, g.926T>G as ss107796307, g.1052G>A as rs1800450, g.1061G>A as rs1800451. All ss numbers were submitted by the authors to the SNP database and will be changed to rs numbers in the near future. [file 1475-2875-8-97-S1.doc]

Additional file 1 - *MBL2* nucleotide changes and haplotype frequencies in the Gabonese samples according to parasitaemia status

|  | **273** | **388** | **396** | **474** | **478** | **482** | **487** | **495** | **578** | **602** | **659** | **712** | **753** | **797** | **826** | **925** | **926** | **1052** | **1061** | **PCR-**  ***N= 124** | **PCR+**  **N=152** | **PCR+TBS+**  **N = 34** |
| --- | --- | --- | --- | --- | --- | --- | --- | --- | --- | --- | --- | --- | --- | --- | --- | --- | --- | --- | --- | --- | --- | --- |
| *LYPA* | C | G | A | A | G | A | A | AAAGAG | G | G | C | A | C | C | C | C | T | G | G | 16.94 | 13.2 | 8.82 |
| *LYPAs1* | . | A | . | . | . | . | . | . | . | . | . | . | . | . | . | . | . | . | . | 5.65 | 8.55 | 5.88 |
| *LYPAs2* | . | . | . | . | A | . | . | . | . | . | . | . | . | . | . | . | . | . | . | 0.81 | 0.66 | 0 |
| *LYPAs3* | . | . | . | . | . | . | G | . | . | . | . | . | . | . | . | . | . | . | . | 0 | 0.66 | 0 |
| *LYPF* | . | . | . | . | . | . | . | . | . | . | . | . | . | . | . | G | G | . | . | 0 | 0.66 | 2.94 |
| All *LYPA* |  |  |  |  |  |  |  |  |  |  |  |  |  |  |  |  |  |  |  | 23.4 | 23.73 | 17.64 |
| *HYPA* | G | . | . | . | . | . | . | . | . | . | . | . | . | . | . | . | . | . | . | 4.84 | 3.95 | 8.82 |
| *LXPA* | . | . | . | . | . | . | . | . | . | C | . | . | . | . | . | . | . | . | . | 15.32 | 13.8 | 5.88 |
| *LYPB* | . | . | . | . | . | . | . | . | . | . | . | . | . | . | . | . | . | A | . | 2.42 | 3.29 | 0 |
| *LYQA* | . | . | C | G | . | . | G | ------ | . | . | . | . | T | . | T | . | . | . | . | 28.22 | 24.3 | 23.53 |
| *LYQAs1* | . | . | C | G | . | . | G | ------ | . | . | T | . | T | . | T | . | . | . | . | 0.81 | 0 | 0 |
| *LYQAs2* | . | . | C | G | . | . | G | ------ | A | . | . | . | T | . | T | . | . | . | . | 6.45 | 8.55 | 8.82 |
| *All LYQA* |  |  |  |  |  |  |  |  |  |  |  |  |  |  |  |  |  |  |  | 37.9 | 36.14 | 32.35 |
| *LYQC* | . | . | C | G | . | . | G | ------ | . | . | . | . | T | . | T | . | . | . | A | **13.71** | 18.4 | **29.41** |
| *LYQCs1* | . | . | C | G | . | . | G | ------ | . | . | . | . | T | A | T | . | . | . | A | 2.42 | 3.29 | 2.94 |
| *LYQCs2* | . | . | C | G | . | G | G | ------ | . | . | . | . | T | A | T | . | . | . | A | 0.81 | 0 | 0 |
| *LYQCs3* | . | . | C | G | . | . | G | ------ | . | . | . | T | T | . | T | . | . | . | A | 1.61 | 0.66 | 2.94 |
| *All LYQC* |  |  |  |  |  |  |  |  |  |  |  |  |  |  |  |  |  |  |  | 18.55 | 22.35 | 35.29 |
